# The Curve of Health

**Published:** 1891-03

**Authors:** 


### The Curve of Health.

Oliver Wendell Holmes says in the *Atlantic Monthly:* Let me tell you one thing. I think if patients and physicians were in the habit of recognizing the fact that I am going to mention both would be gainers. The law I refer to must be familiar to all observing physicians, and to all intelligent persons who have observed their own bodily and mental conditions. This is, the curve of health. It is a mistake to suppose that the normal state of health is represented by a straight horizontal line. Independently of the well-known causes which raise or depress the standard of vitality, there seems to be—I think I may venture to say there is—a rhythmic undulation in the flow of the vital force. The "dynamo" which furnishes the working powers of consciousness and action has its annual, its monthly, its diurnal waves, even its momentary ripples, in the current it furnishes. There are greater and lesser curves in the movement of every days's life—a series of ascending and descending movements, a periodicity depending on the very nature of the force at work in living organism. Thus we have our good seasons and our bad seasons, our good days and our bad days, climbing and descending in long or short undulations, which I have called the curve of health. From this fact springs a great proportion of the errors of medical practice. On it are based the delusions of the various shadowy systems which impose themselves on the ignorant and half-learned public, as branches or "schools" of science. A remedy taken at the time of the ascent in the curve of health is found successful. The same remedy taken while the curve is in its downward movement proves a failure. So long as this biological law exists, so long the charlatan will keep his hold on the ignorant public. So long as it exists the wisest practitioner will be liable to deceive himself about the effect of what he calls, and loves to think are, his remedies. Long-continued and sagacious observation will, to some extent, undeceive him; but were it not for the happy illusion that his useless or even deleterious drugs were doing good service, many a practitioner would give up his calling for one in which he could be more certain that he was really doing good to the subjects of his professional dealings.

